# Dysfunction and regulatory interplay of T and B cells in chronic hepatitis B: immunotherapy and emerging antiviral strategies

**DOI:** 10.3389/fcimb.2024.1488527

**Published:** 2024-12-09

**Authors:** Fei Yu, Yue Zhu, Shenghao Li, Liyuan Hao, Na Li, Fanghang Ye, Zhi Jiang, Xiaoyu Hu

**Affiliations:** ^1^ School of Clinical Medicine, Chengdu University of Traditional Chinese Medicine, Chengdu, Sichuan, China; ^2^ Department of Infectious Diseases, Hospital of Chengdu University of Traditional Chinese Medicine, Chengdu, Sichuan, China

**Keywords:** hepatitis B virus, immune tolerance, immunotherapy, antiviral therapy, B cells, T cells

## Abstract

In the context of chronic hepatitis B virus (HBV) infection, the continuous replication of HBV within host hepatocytes is a characteristic feature. Rather than directly causing hepatocyte destruction, this replication leads to immune dysfunction and establishes a state of T-B immune tolerance. Successful clearance of the HBV virus is dependent on the close collaboration between humoral and cellular immunity. Humoral immunity, mediated by B-cell subpopulations, and cellular immunity, dominated by T-cell subpopulations show varying degrees of dysfunction during chronic hepatitis B (CHB). Notably, not all T- and B-cells produce positive immune responses. This review examine the most recent developments in the mutual regulation of T-B cells during chronic HBV infection. Our focus is on the prevailing immunotherapeutic strategies, such as T cell engineering, HBV-related vaccines, PD-1 inhibitors, and Toll-like receptor agonists. While nucleos(t)ide analogues (NUCs) and interferons have notable limitations, including inadequate viral suppression, drug resistance, and adverse reactions, several HBV entry inhibitors have shown promising clinical efficacy. To overcome the challenges posed by NUCs or monotherapy, the combination of immunotherapy and novel antiviral agents presents a promising avenue for future CHB treatment and potential cure.

## 1 Introduction

Hepatitis B virus (HBV) infection represents a significant global health threat. According to WHO, in 2019, there were 296 million people worldwide diagnosed with chronic hepatitis B (CHB), and 1.5 million new infections were reported each year (World Health Organization, 2023). The consequences of this infection can be severe, potentially culminating in chronic hepatitis, cirrhosis, and even hepatocellular carcinoma (HCC) (Liang, 2009). Most HBV infections lead to acute self-limiting diseases. More than 90% of infected adults recuperate under a robust immune response and with the subsequent disappearance of HBsAg and seroconversion of anti-HBs, the virus gets eradicated, yielding immunity (Pollicino and Caminiti, 2021). The host immune response plays a crucial role in interpreting the clinical outcomes of hepatitis B virus (HBV) infections, determining whether the infections are cleared or progress into chronic infections (Jose-Abrego et al., 2023). The American Association for the Study of Liver Diseases (AASLD) defines chronic infection as the presence of HBsAg for at least six months (Conners et al., 2023). The three primary serological markers used to determine the status of HBV infection are Hepatitis B surface Antigen (HBsAg), Anti-Hepatitis B surface (Anti-HBs), and Anti-Hepatitis B core (Anti-HBc). During the acute phase of an HBV infection, HBsAg, Anti-HBc, and IgM Anti-HBc persist. As the hosts’ immune response effectively eliminates the virus, the levels of HBsAg gradually decrease and the presence of Anti-HBs signifies recovery from HBV infection. In the typical process of chronic infection, both HBsAg and anti -HBc will appear, while IgM anti -HBc will disappear (Conners et al., 2023).

This highlights the critical significance of addressing and managing HBV to mitigate its impact on public health. Currently, nucleoside (acid) analogs (NUCs) and pegylated interferon (Peg-IFN) are two approved treatments for chronic HBV infection (Tang et al., 2018; Terrault et al., 2018; Xia and Liang, 2019). The first-line approach involving NUCs effectively suppresses viral replication, leading to improved clinical outcomes. Long-term antiviral therapy with NUCs has demonstrated the potential to further reduce the threat of progression to cirrhosis and HCC (Trépo et al., 2014; Yuen et al., 2018). Despite the efficacy of NUCs in inhibiting viral replication, achieving a functional cure, characterized by persistent HBsAg negativity presence/absence of HBsAb, and undetectable HBV-DNA in serum remains challenging. This challenge is ascribed to the presence of covalently closed circular DNA (cccDNA) in hepatocytes. The existence of cccDNA poses a hurdle to achieving a sustained virologic response, potentially leading to virologic relapse after discontinuation of the drug (Ramji et al., 2022). On the other hand, Peg-IFN, being the sole approved immunomodulatory drug, is characterized by a low response rate and often comes with intolerable side effects (Ghany, 2017). One of the critical challenges in achieving a cure for HBV infection is the presence of HBV cccDNA. This persistent viral reservoir has a crucial function in sustaining HBV infection and acts as a key barrier to the successful eradication of the disease (Xia and Guo, 2020).

Chronic HBV infection is an intricate and dynamic process that involves HBV, hepatocytes, host immune system, liver injury and viral control, and clinical regression that depends on the complex interaction between viral replication and host immune response. The natural course of CHB can be categorized into four distinct phases, each characterized by specific features (Liaw and Chu, 2009; Liaw, 2019): immune tolerance phase (elevated HBV-DNA, normal ALT levels, and HBeAg positivity), immune clearance phase (high HBV-DNA, heightened ALT levels with active hepatic inflammation, and HBeAg positivity), immune control phase (HBV-DNA <2×10^3^ IU/mL, normal ALT levels and HBeAg negativity), and reactivation phase (HBV-DNA ≥2×10^3^IU/mL, ALT persistently or repeatedly elevated). Throughout the persistent HBV infection, the immune response of the host to the virus experiences varying degrees of dysfunction. Understanding these distinct phases is crucial for comprehending the evolving nature of chronic HBV infection and for tailoring appropriate therapeutic interventions at various disease stages.

Recently, research has consistently detected the presence of CD8^+^ T-cell exhaustion in patients with chronic HBV infection and tumors. The gradual dysfunction or outright loss of function observed in T-cells is attributed to prolonged exposure to persistent viral antigens and inflammatory stimuli. Depleted T-cells often express diverse inhibitory receptors (IRs), including but not limited to programmed death receptor 1 (PD-1), cytotoxic T-lymphocyte-associated protein 4 (CTLA-4), lymphocyte activation gene 3 (LAG-3), T-cell immunoglobulin structural domain and mucin structural domain 3 (TIM-3), among others. The co-expression and upregulated expression of IRs on the surface of T cells represent a vital feature linked to T-cell exhaustion. Therefore CD8^+^ T-cell exhaustion has gradually become a focus for exploring the mechanism of chronic HBV infection (Ye et al., 2015; Fisicaro et al., 2020; Allahmoradi et al., 2023; Li et al., 2023). B cells and T cells as important elements of human adaptive immunity are closely linked during chronic HBV infection. Their collaboration involves various crucial functions such as the secretion of antibodies and cytokines, the presentation of antigens on the cell surface, and immune cell activation. Together, B cells and T cells play pivotal roles in mediating chronic HBV infection, immune tolerance, and liver injury. However, in the context of chronic HBV infection, there has been limited research on the immune interactions between B cells and T cells. Considerable research has been performed to identify the targets for HBV-associated T-cell failure, but few studies focused on internal B cell defect. Clinical evidence is increasingly supporting the involvement of B cells in the immune control of HBV. The role of B cells and their protective antibodies is equally significant in the context of chronic HBV infection (Barouch et al., 2013; Shingai et al., 2013; Zhong et al., 2022). This research dealt with a comprehensive review of the latest studies of immunodeficiency and dysfunction with a specific focus on B and T cells during chronic HBV infection. Additionally, we summarized some of the immunotherapeutic strategies currently applied in clinical practice and discussed future immunotherapeutic strategies, hoping to provide new insights for immunotherapy protocols.

## 2 B-cell subsets and T-cell subsets in persistent HBV infection

When HBV infects host cells, the virus can rapidly stimulate cellular pattern recognition receptors (PRRs) and host innate immunity is activated as the first line of defense against HBV infection (Yang et al., 2022). However, HBV is considered to be a “stealth virus” that evades recognition by the host innate immune system and establishes chronic infection in hepatocytes. The Na+/taurocholate cotransporting polypeptide (NTCP) is a receptor commonly expressed on the surface of hepatocytes and serves as a specific entry receptor for HBV and HDV infection. The initial low-affinity binding between HBV and heparin sulfate proteoglycans, such as glypican 5, on the liver cell membrane initiates viral entry. Subsequently, the N-terminal region of the preS1 antigen on the surface of HBV binds to NTCP on the liver cell surface and enters the cell through clathrin-mediated endocytosis (CME) (Verrier et al., 2016; Herrscher et al., 2020; Wei and Ploss, 2021). The epidermal growth factor receptor (EGFR) plays a crucial role in facilitating the internalization of HBV-NTCP complexes into hepatocytes (Iwamoto et al., 2019; Iwamoto et al., 2020).

**Figure 1:**
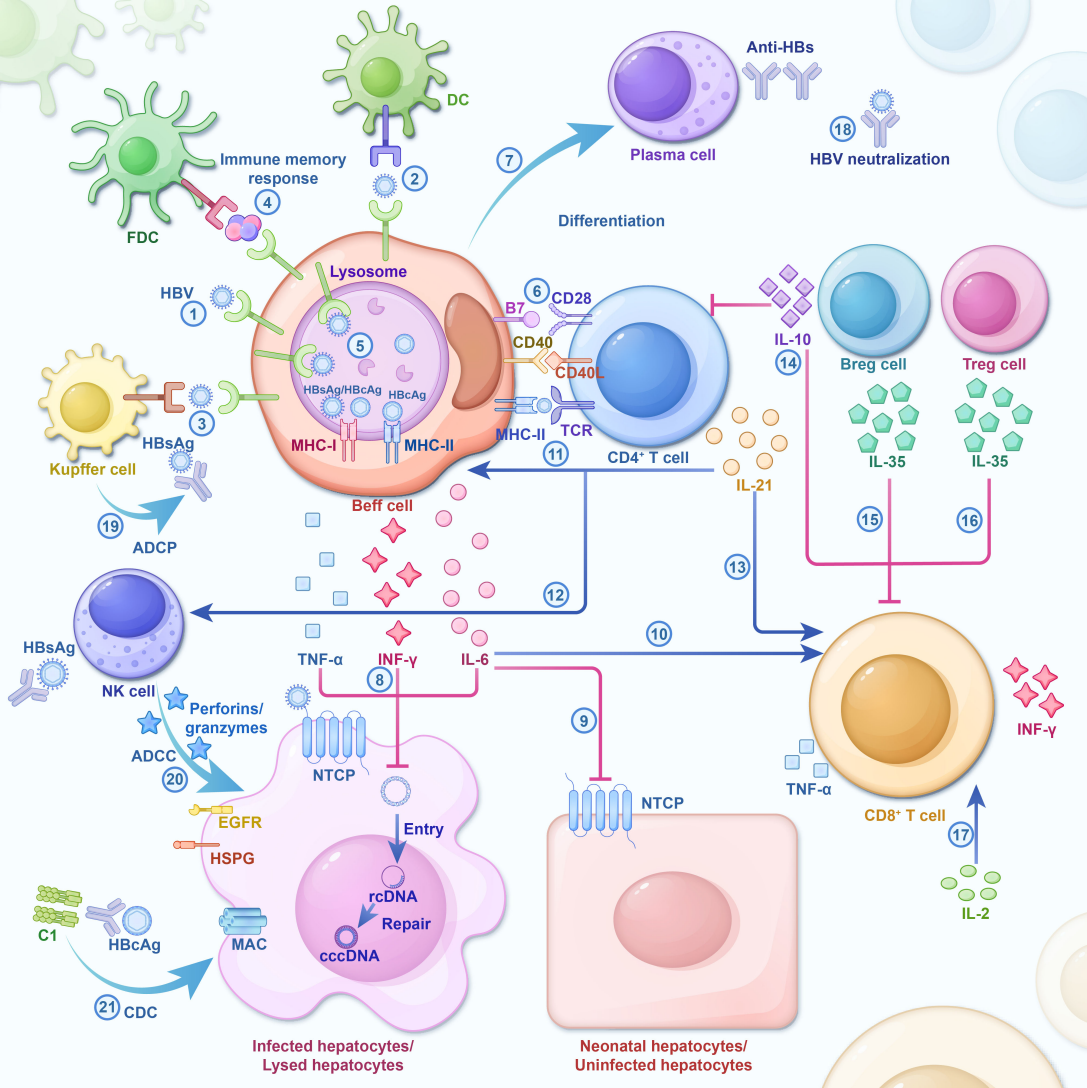
During HBV infection, cells and significant cellular factors are activated and form an interaction network. The host immune response is a key driver of HBV clearance, and its absence ensures chronic infection. As a result of the strong and multi-specific anti-viral immune response, most adults spontaneously recover from acute infections post-HBV exposure. However, some inhibitory cellular factors involved in the exhaustion of HBV-specific immune cells during chronic infection impede effective virus clearance. HBV infection triggers the initial cascade of cytokines (Liang, 2009; Pollicino and Caminiti, 2021; World Health Organization, 2023): independent HBV antigens or HBV antigens on Kupffer cells or DC surfaces, which bind after being specifically recognized by BCR (Jose-Abrego et al., 2023). Immunocomplex sacks on FDC dendrites potentially participating in B cell’s immune memory response (Conners et al., 2023). HBV antigen is catabolized and processed by the B cells to HBcAg/HBsAg antigen, and in the lysosome, MHC-I/MHC-II molecules produced by the B cells are combined to form HBcAg-MHC-II complex and HBcAg/HBsAg-MHC-I complex and then transferred to the surface of the plasma membrane. Immune cell activation and cytokine secretion (Xia and Liang, 2019): Upon induction by antigen and co-stimulatory signals, Beff cells recruit CD4+ T cells, resulting in the activation, proliferation, and differentiation of both Beff and CD4^+^ T cells (Terrault et al., 2018). Activated CD4^+^T cells secrete IL-21 to induce the differentiation of Beff cells into PCs (Tang et al., 2018). B-Cells produce IL-6, IFN-γ, and TNF-α to catalyze cccDNA decay in Hepatocytes infected by HBV (Trépo et al., 2014). IL-6 restricts the intrusion of HBV into normal hepatocytes by understating the expression of NTCP (Yuen et al., 2018). IL-6 enhances the immunological response of T cells (Ghany, 2017; Xia and Guo, 2020; Ramji et al., 2022). IL-21 promotes the proliferation and differentiation of Beff cells, CD4^+^ T cells, and NK cells, meanwhile strengthening the cytotoxicity of CD8^+^ T cells and NK cells (Liaw and Chu, 2009; Liaw, 2019; Allahmoradi et al., 2023). Breg cells and Treg cells secrete anti-inflammatory cytokines (e.g., IL-10, IL-35) that inhibit the proliferation and effector functionality of T cells (Li et al., 2023). IL-2, an autocrine cytokine produced by activated T cells, can stimulate T cell self-proliferation, survival, and effectual differentiation. The Immune Response Mediated by Anti-HBs (Ye et al., 2015): Plasma cells secrete a large amount of Anti-HBs which directly neutralizes HBV (Fisicaro et al., 2020). Anti-HBs binds to HBsAg and prompts the Kupffer cells to carry out ADCP (Barouch et al., 2013). Anti-HBs binds to HBsAg and stimulates NK cells to release Perforin/Granzymes, resulting in ADCC, which depletes infected liver cells (Shingai et al., 2013). Anti-HBc IgG binds to HBcAg and induces the lysis of infected liver cells via CDC. DC, Dendritic cell; FDC, Follicular dendritic cells; NK cell, Natural killer cell; BCR, B-cell receptor; TCR, T-cell receptor; MAC, Membrane Attack Complex; HSPG, heparin sulfate proteoglycans; NTCP, Na+/taurocholate Cotransporting Polypeptide; Beff cells, Effector B cell; Breg cell, regulatory B cell; Treg cell, regulatory T cell; PC, plasma cell; ADCP, antibody-dependent cellular phagocytosis; ADCC, antibody-dependent cytotoxicity; CDC, complement-dependent cytotoxicity; Plain arrows indicate inhibitory effects and sharp arrows indicate enhanced or promoting effects.

A partial double-stranded relaxed circular DNA (rcDNA) containing the HBV genome is transported to the host hepatocyte nucleus. Subsequently, the biogenesis of cccDNA is completed through three steps: nuclear transport of rcDNA, rcDNA repair, and cccDNA chromatinization (Jiang and Hildt, 2020; Wei and Ploss, 2021). cccDNA serves as a template for transcription to initiate HBV gene expression and replication. During chronic HBV infection, HBV-specific T cells and B cells demonstrate varying degrees of immunological dysfunction, hindering the effective eradication of the virus (Ma et al., 2019; Cai and Yin, 2020; Fisicaro et al., 2020; Baudi et al., 2021). Several mechanisms contribute to this immunotolerant microenvironment in the liver, including the downregulation of co-stimulatory molecule expression, the negative signaling transmission of key immune cells and inhibitory cell factors. This facilitates the persistence of HBV infection, as shown in **Figure 1**.

### 2.1 B-cell antigenic peptide presentation process

**Figure 2:**
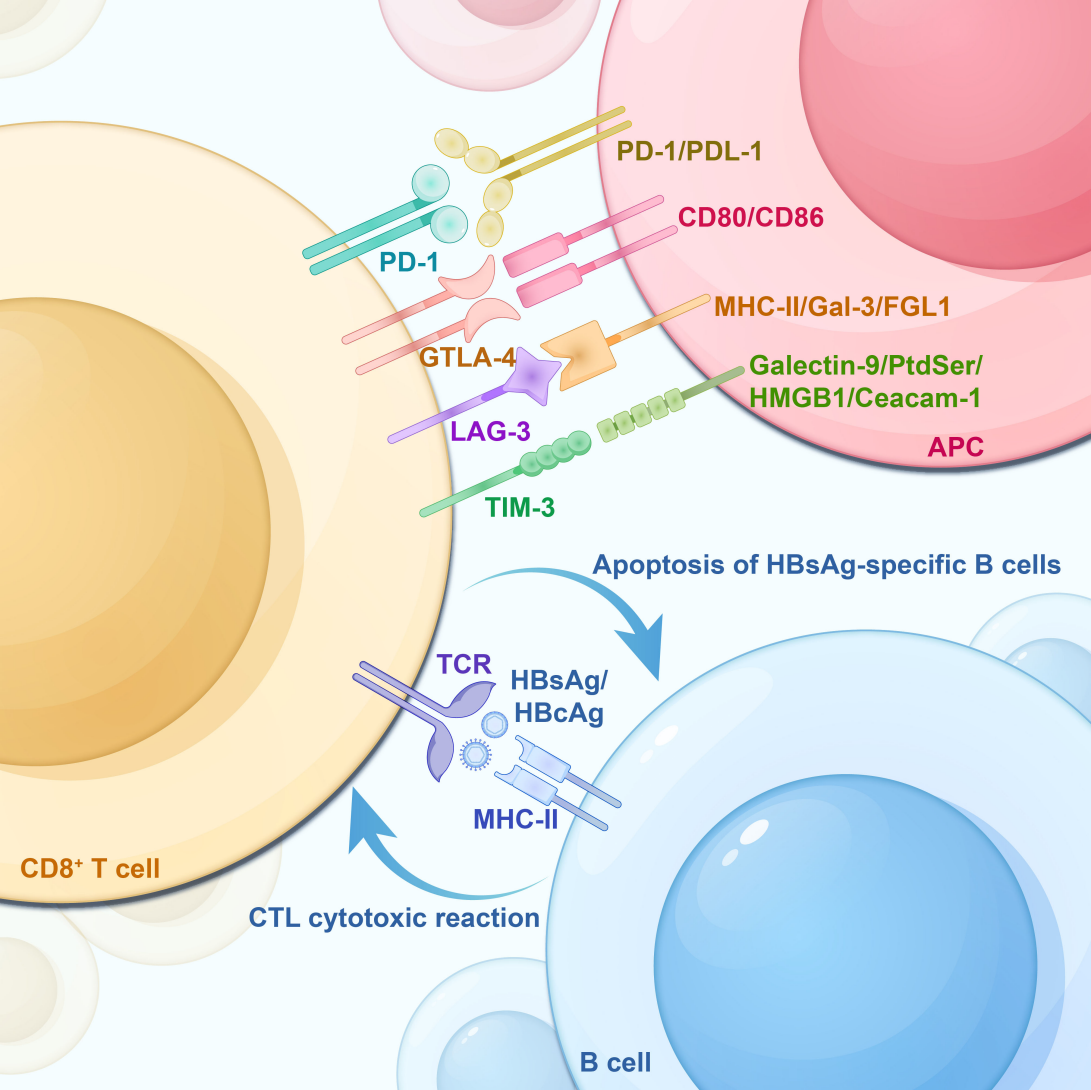
HBsAg-specific B-cell apoptosis, T-cell exhaustion -associated IRs and their corresponding ligands on APCs. CD8^+^ T cells are activated by the HBcAg/HBsAg-MHC-I complex on the surface of B cells, inducing cytotoxic T lymphocyte (CTL) to produce a cytotoxic response. However, this process backfires on the HBsAg-specific B cells, leading to their apoptosis. IRs, Inhibitory Receptors; TCR, T-cell receptor; PD-1, Programmed Death Receptor 1; Tim-3, T Cell Immunoglobulin and Mucin Structural Domain Molecules 3; CTLA-4, cytotoxic T-lymphocyte-associated protein 4; LAG-3, lymphocyte activation gene 3; APC, antigen-presenting cell; PD-L1, programmed death-ligand 1; PD-L2, programmed death-ligand 2; Galectin-9, galactoglucan lectin 9; PtdSer, phosphatidylserine; HMGB1, high-mobility group protein B1; Ceacam-1, carcinoembryonic antigen-associated cell adhesion molecule 1.

As professional antigen-presenting cells (APCs), B cells use their receptors (BCRs) to specifically recognize HBV antigens on the surface of Kupffer cells or dendritic cells (DCs). Following antigen recognition, the BCR-antigen complexes are internalized into endosomes via receptor-mediated endocytosis, where B cells subsequently process these antigens and present them on MHC-I and MHC-II molecules within lysosomes. These molecules then bind to processed HBcAg or HBsAg antigens, forming HBcAg-MHC-II and HBsAg/HBcAg-MHC-I complexes, respectively. Subsequently, these complexes are transported to the plasma membrane surface (Cai and Yin, 2020). Simultaneously, under the activation signals from multiple pairs of co-stimulatory molecules, including B7 and CD28, CD40 with CD40L, naïve CD4^+^ T cells are activated by B cells presenting the HBcAg-MHC-II complex. This activation initiates a CD4^+^ T cell immune response. However, CD8^+^ T cells are activated by B cells presenting the HBcAg/HBsAg-MHC-I complex, collectively inducing a Cytotoxic T-lymphocytes (CTLs) cytotoxic response (Barnaba et al., 1990; Gehring et al., 2013; McIntosh et al., 2014; Cai and Yin, 2020; Sajjad et al., 2021). In addition, follicular dendritic cells (FDCs) may participate in specialized antigen presentation, a pathway linked to the formation of memory B cells and plasma cells(PCs). FDCs are a class of cells that line the antigen-exposed surfaces of B-cell follicles in lymph nodes and spleens. These cells engage in close interactions with B cells, express Fc receptors, and possess the unique ability to preserve antigen-antibody complexes for an extended duration. This preservation is achieved through the formation of immune complex-encapsulated vesicles on their dendrites. FDCs may participate in the immune memory response of B cells, which may take up antigens from FDCs and process and present them to T cells (MacLennan, 1994; Heath et al., 2019). However, low expression of the co-stimulatory molecule CD40 on B cells of patients with chronic HBV infection may affect the co-stimulation between CD40 and CD40L, thus suppressing the growth and activation of B cells (Xing et al., 2013). Furthermore, the surface of B cells in patients with CHB also lacks the expression of the co-stimulatory molecule CD80 (Xu et al., 2015). As a key type of APCs, B cells may also be implicated in T cell damage during chronic HBV infection. The research by Barnaba et al. showed that HBsAg-specific B cells cross-present HBsAg fragments to CTLs via the MHC-I molecule. Nevertheless, the induction of cytotoxicity in CTLs during this process might lead to the death of HBsAg-specific B cells (Barnaba et al., 1990; Keller et al., 2009), as shown in **Figure 2**.

### 2.2 Positive antiviral immune response generated by effector B cells and effector T cells

B cells form a germinal center by recruiting specific CD4^+^ T cell helpers. This process stimulates the proliferation and differentiation of B cells following induction by antigenic signals, involving the identification of HBV peptide-MHC-class II molecular complexes on the surface of B cells by Th cells. Co-stimulatory signals, facilitated by the interaction of CD40 and B7 on the B cell surface with various co-stimulatory molecules on the Th cell surface (including CD40L and CD28), further contribute to this immune response. The CD4^+^ T cell receptor (TCR) on T cell surfaces binds to HBV antigenic peptide-MHC-class II molecular complexes. Non-specific co-stimulatory signals, facilitated by co-stimulatory molecules like B7 and CD40, activate CD4^+^ T cells, leading them into a proliferative and differentiated state. Notably, the co-stimulatory molecule B7 is essential for T cell activation and differentiation, with interactions between B7 and CD28 playing a key role in delivering essential stimulatory signals to T cells (Azuma et al., 1993; Freeman et al., 1993; Janakiram et al., 2017). Anti-HBs are secreted by B cells and act as a protective antibody to directly neutralize pathogens and block HBV replication and its entry into hepatocytes (Lu et al., 2018). Additionally, anti-HBs also fulfill other multi-effect functions during chronic HBV infection. Immunohistological studies have shown a significant correlation between immune-mediated hepatitis injury during chronic HBV infection and hepatocyte membrane-associated HBsAg. It is suggested that membrane HBsAg could potentially act as a target antigen for T cell-mediated immune lysis of hepatocytes (Ray et al., 1976; Huang and Neurath, 1979). The finding of cytoplasmic/membrane HBsAg on infected hepatocytes showed that anti-HBs IgG may bind HBsAg, triggering the release of perforin/granzyme in NK cells. This activation results in antibody-dependent cytotoxicity (ADCC), leading to the exhaustion of HBV-infected hepatocytes (Chu and Liaw, 1987; Ackerman et al., 2016; Lu et al., 2018). Theoretically, anti-HBs also bind to HBsAg and induce Kupffer cells to engage in antibody-dependent cellular phagocytosis (ADCP) and contribute to the immune clearance process of HBV (Lu et al., 2018). Beyond the induction of NK cells for ADCC-mediated lysis of hepatocytes infected with HBV, driven by membrane-associated HBsAg and HBcAg, contributes to hepatocyte injury through complement system activation. In patients with chronic HBV infection, elevated levels of anti-HBc IgG in the liver may bind to HBcAg, leading to extensive formation of antigen-antibody complexes on the surfaces of hepatocytes and Kupffer cells. This further activates the classical complement pathway and complement system through a series of zymogenic cascade reactions. Ultimately, this culminates in the assembly of membrane attack complexes (MACs) to lyse virus-infected hepatocytes and cause hepatocyte necrosis (Farci et al., 2010; Mutti et al., 2018).

T follicular helper (Tfh) cells play a role in regulating regulatory B (Breg) cells HBV-associated humoral immune response (Hu et al., 2014). IL-21 is a cytokine primarily released by activated CD4^+^ T cells (primarily Tfh cells and Th17 cells). It plays a crucial role in sustaining the effector response of CD8^+^ T cells during chronic viral infections (Zander et al., 2022). HBV antigenic peptides are presented to CD4^+^ T cells via MHC-II, and these interactions stimulate IL-21 production by CD4^+^ T cells, especially Tfh (Asao, 2021). IL-21 can induce the differentiation of naive and memory B cells into PCs, leading to substantial production of IgG antibodies. Moreover, IL-21 plays a role in regulating the differentiation, proliferation, and activation of T cells, B cells, and NK cells (Ettinger et al., 2005; Shbeer and Ahmed Robadi, 2023). IL-21 produced by Tfh cells also elevates the number of IgG-expressing B cells and stimulates the diversification of HBV-specific T-cell responses. This includes an enhancement in the production of IFN-γ (Publicover et al., 2011). Beyond its involvement in promoting the proliferation, differentiation, and activation of B and CD4^+^ T cells, IL-21 is also capable of augmenting the cytotoxicity of CD8+ T and NK cells, along with increasing IFN-γ production (Yi et al., 2010). The hallmark of T cell senescence in humans is the absence of CD28 expression. IL-21 exhibits the capability to enhance the growth of CD28(+) CD8^+^ T cells by preventing the downregulation of CD28 (Alves et al., 2005; Nguyen and Weng, 2010; Wang et al., 2020). Furthermore, IL-21 can directly act on CD8^+^ T cells to maintain CD8^+^ T cell responsiveness in chronic viral infections (Yi et al., 2010). Also, IL-21 inhibits Foxp3+ regulatory CD4^+^ T cells, resulting in a reduction in the number of suppressor regulatory T cells (Tregs), which indirectly promotes the production of antigen-specific CD8^+^ cytotoxic T lymphocytes (Li and Yee, 2008). Recent research indicated the capacity of IL-21 to promote the generation of HBV-specific CD8^+^ T cells while downregulating the expression of PD-1 and TIM-3. This observation highlights that IL-21 could contribute to the clearance of HBV (Tang et al., 2019). These prior outcomes indicated that the pleiotropic effects of IL-21 exert a positive immune influence during viral infection.

CTLs also kill HBV-infected target cells by secreting cytokines, including IFN-γ and TNF-α. IFN-γ has a pleiotropic effect. IFN-γ possesses immunomodulatory properties, activating the functions of NK and T cells, along with macrophages. This activation helps regulate the immune microenvironment, applying immune pressure on infected hepatocytes. Simultaneously, IFN-γ impedes HBV replication through a non-cytolytic mechanism by inhibiting HBV transcription (Nosaka et al., 2020; Chen et al., 2021). However, T-cell exhaustion often occurs during persistent HBV infection, and cytokines (e.g., IL-2 and IL-12) can antagonize T-cell exhaustion. IL-2, primarily produced by activated T cells, functions as an autocrine cytokine that facilitates self-activation and proliferation. It is often referred to as the T cell growth factor. The signaling of IL-2 is closely linked to both antigen presence and co-stimulation, and the interaction of these two factors propels T-cell proliferation, sustains cell survival, and guides effector differentiation. Additionally, IL-2 is critically involved in the formation of long-lived CD8^+^ T cell memory (Abbas et al., 2018; Kalia and Sarkar, 2018). By receiving IL-2, virus-specific CD8^+^ T cells that were previously impaired regain their capacity for growth, differentiation, and IFN-γ production (Bénéchet et al., 2019). Furthermore, IL-12, a cytokine produced by activated APCs(e.g., dendritic cells, macrophages, etc.), serves as a potent inducer of IFN-γ production by T and NK cells. The activation of CD8^+^T cells requires a variety of signals. In addition to the antigen signal generated by TCR recognizing antigen peptide -MHC-I molecular complex and the co-stimulation signal generated by several pairs of co-stimulatory molecules (mainly CD28 and B7), pro-inflammatory cytokines IL-12 and/or IFN-α could also serve as the third signal of T cell activation to jointly promote the proliferation and differentiation of CD8^+^T cells. The activation of CD8^+^T cells requires a variety of signals. In addition to the antigen signal generated by TCR recognizing antigen peptide -MHC-I molecular complex and co-stimulation signal generated by several pairs of co-stimulatory molecules (mainly CD28 and B7), researchers gradually realize that pro-inflammatory cytokines IL-12 and/or IFN-α can be used as the third signal for T cell activation. This third signal collaborates to enhance the proliferation and differentiation of CD8^+^T cells (Curtsinger and Mescher, 2010). IL-12 is capable of down-regulating the expression of PD-1 on HBV-specific T cells, thereby restoring a diverse and multifunctional CD8^+^ T cell response in order to enhance cytotoxicity (Schurich et al., 2013). Moreover, Schurich’s team found that IL-12 can correct mitochondrial metabolic disorders in dysfunctional CD8^+^ T cells, enhance their mitochondrial potential, and reduce their reliance on glycolysis, thereby restoring HBV-specific T cell effector function (Schurich et al., 2016).

### 2.3 Two subpopulations of B cells: effector B cells and regulatory B cells

In addition to producing antibodies, B lymphocytes contribute to immune system activation through cytokine production and antigen presentation. B cells can be sorted into subpopulations as per their functional characteristics, including effector B (Beff) cells and Breg cells (Matsushita, 2019). Breg cells exhibit a dual function by exerting both negative regulatory immune responses and protective immune responses via the production of anti-inflammatory cytokines, including IL-10, IL-35, and transforming growth factor-β (TGF-β) (Matsushita, 2019). In contrast, Beff cells secrete cytokines, including IL-6, IFN-γ, and tumor necrosis factor-α (TNF-α), to exert pro-inflammatory immune responses and enhance responses of effector cells and memory CD4^+^ T cells (Shen and Fillatreau, 2015; Matsushita, 2019). These pro-inflammatory cytokines may induce cccDNA degradation, inhibit HBV replication and cccDNA transcription, thereby reducing the level of HBV cccDNA in hepatocytes and exerting a non-cytolytic antiviral effect on infected hepatocytes (Hösel et al., 2009; Palumbo et al., 2015; Xia et al., 2016). IL-6 has the capability to hinder the entry of HBV into hepatocytes by suppressing the expression of NTCP. Downregulated local NTCP in the hepatocyte membrane confers neonatal hepatocytes resistance to HBV reinfection, which may accelerate virus clearance during immune-mediated cell death and compensatory proliferation of living hepatocytes (Yan et al., 2012; Bouezzedine et al., 2015; Yan et al., 2019).

In chronic HBV infection, most Breg cells are capable of expressing the anti-inflammatory cytokine IL-10. This expression enhances the cellular function of Tregs while concurrently suppressing the immune function of effector T cells to maintain immune tolerance (Mauri and Bosma, 2012; Liu et al., 2016). In CHB patients, B cells that produce IL-10 primarily display immature/transitional phenotypes characterized by CD19 ^+^ CD24hi CD38hi expression. IL-10 secreted by Breg cells inhibits HBV-specific CD8^+^ T cell responses. Notably, the suppression can be restored after the exhaustion of Breg cells (Das et al., 2012). Bregs also have an impact on the activated CD4^+^ T cells. A study reveals that IL-10 secreted by Bregs exerts a negative regulatory effect on the T cell-dependent inflammatory response. This secretion inhibits both the proliferation of effector CD4^+^ T cells and the activation of antigen-specific CD8^+^ T cells (Catalán et al., 2021). Similarly, another experiment on IL-10-deficient mice shows the same conclusion. Compared with the new IL-10 transcription reporter mice, the B-cell-specific IL-10-deficient mice exhibit an increased specific CD8^+^ T cell response to murine cytomegalovirus and an elevation in the number of PCs (Madan et al., 2009).

It is crucial to emphasize that IL-35, another immunosuppressive factor secreted by Tregs and Breg cells, has the capacity to inhibit T-cell proliferation and effector functions (Xiang and Xie, 2015). Interestingly, IL-35, which exhibits elevated expression levels in CD4^+^ T cells from patients with chronic HBV infection, demonstrates the ability to suppress HBV-specific CTL cell proliferation and IFN-γ production *in vitro* (Li et al., 2015). In addition, it was found that IL-35 played a key function in various diseases, including inflammatory bowel disease (Li et al., 2014), autoimmune diabetes (Bettini et al., 2012), T-cell-dependent colitis (Wirtz et al., 2011), human prostate tumors (Olson et al., 2012), colorectal cancer (Zeng et al., 2013), and systemic lupus erythematosus (Okamoto et al., 2011). IL-35 can modulate T-cell-mediated specific immune responses (Collison et al., 2007; Okamoto et al., 2011; Wirtz et al., 2011; Bettini et al., 2012; Olson et al., 2012).

### 2.4 B-cell functional impairment

B cells are critically involved in the immune control of HBV through their antibody secretory function and immunomodulatory effects, etc. During chronic HBV infection, varying degrees of B cell immunodeficiency were observed, a phenomenon that impedes HBV clearance. A lack of HBsAg-specific B cells and significantly lower plasma anti-HBs levels in CHB patients could be partially restored in HBsAg seroconversion responders, which confirms the important roles of HBsAg-specific B cells and anti-HBs during HBV clearance (Xu et al., 2015). The acquired data suggest that the functional impairment observed in HBsAg-specific B cells, particularly in their ability to secrete anti-HBs antibodies, may contribute to the persistence of HBV infection. In addition, Breg cells and their secreted IL-10 and IL-35 also play a role in immunomodulating and maintaining immune tolerance by enhancing the function of Treg cells and inhibiting the immune function of effector T cells. While research on Breg cell-derived IL-35 in HBV infection is limited, accessed data from previous research indicated the immunosuppressive role of IL-35 in immune-related diseases. Based on this, we speculated that IL-35 played a vital role in the immune-mediated hepatic injury and immune tolerance observed throughout chronic HBV infection. Specifically, it is likely involved in suppressing the proliferation and differentiation of effector T cells. However, this may not be helpful for investigating HBV-associated immunopathogenesis due to the absence of activation and specific marker features on the surface of Breg cells. In pathogen immunity, memory B cells (MBCs) differentiate into antibody-secreting PCs. However, during HBV infection, there are significant alterations observed in peripheral and hepatic B-cell compartments, with HBsAg-specific B cells localizing to the infected liver and atypical memory B cells (atMBCs) preferentially accumulating and up-regulating PD-1 (Burton et al., 2018). The atMBCs present in the overall population of B cells and HBsAg-specific B cells exhibit enrichment in the liver and express a variety of IRs. The atMBC derived from CHB patients shows an attenuated BCR signaling response and reduced ability to secrete important antiviral cytokines such as IL-6 or TNF-α (Burton et al., 2018). Although most studies have focused on the high expression of IRs such as PD-1 on T cells, it is crucial to note that PD-1 is capable of inhibiting B-cell signaling, survival, and activation functions (Okazaki et al., 2001; Good-Jacobson et al., 2010; Titanji et al., 2010; Kardava et al., 2011; Thibult et al., 2013). The binding of PD-1 to BCR recruits tyrosine phosphatase 2 (SHP-2) to the C-terminal phosphotyrosine of PD-1. The subsequently phosphorylated SHP-2 dephosphorylates a key signal transducer of BCR signaling, leading to the inhibition of BCR signaling (Okazaki et al., 2001). Moreover, the collective evidence of reduced production of B-cell protective antibodies, compromised immune responses generated by Bregs, and impaired presentation of HBsAg peptides by B cells due to downregulated expression of co-stimulatory molecules suggests the presence of B-cell functional impairment during chronic HBV infection.

HBV-related vaccines offer potential benefits for addressing B cell dysfunction, with different types serving distinct purposes: preventive vaccines help induce protective antibodies and establish immune memory, while therapeutic vaccines aim to enhance HBV-specific immune responses (Jansen et al., 2021; Mahmood et al., 2023). In a phase 1b clinical trial, the therapeutic vaccine TG1050 demonstrated a favorable safety profile in patients treated for NUC, it was found to elicit HBV-specific cellular immune responses effectively (Zoulim et al., 2020). GS-4774, a novel therapeutic vaccine, encodes three Hepatitis B Virus (HBV) proteins - HBsAg, HBcAg, and HBx. It is designed to stimulate strong and specific T-cell responses against HBV. Current trials indicate that it is both safe and well-tolerated (Gaggar et al., 2014; Lok et al., 2016). Consequently, the suppressive effect of GS-4774 on the virus remains undetermined. A phase 2 clinical trial using GS-4774 showed that though GS-4774 did not reduce the HBsAg levels in chronic HBV-infected patients, it produced a strong immune stimulation effect on the CD8^+^ T cells of vaccinated patients (Boni et al., 2019). The Phase III clinical trial involving a therapeutic vaccine that comprises HBsAg and HBcAg (NASVAC) demonstrates that NASVAC more effectively reduces the viral load to an undetectable level in Chronic Hepatitis B patients when compared to Peg-IFN treatment (Al Mahtab et al., 2018). However, the clinical efficacy and safety of HBV vaccines are influenced by various immunological and clinical factors, and therapeutic vaccines continue to face significant challenges in restoring robust HBV-specific immunity. Consequently, future research will focus on the development of next-generation HBV vaccines.

### 2.5 T-cell dysfunction

#### 2.5.1 Overexpression of immune checkpoints

Persistent high expression and co-expression of immune checkpoints, including PD-1, TIM-3, CTLA-4, and LAG-3, commonly occur in the course of chronic HBV infection due to extended exposure to viral antigens and inflammatory triggers, leading to T-cell exhaustion (Crawford and Wherry, 2009), as shown in **Figure 2**. Among these immune checkpoints, PD-1 is considered a hallmark of CD8^+^ T cell exhaustion (Brooks et al., 2005). PD-L1 and PD-L2 are two known ligands of PD-1. PD-1 inhibitory pathway is involved in the regulation of T cell response during acute and chronic liver inflammation, resulting in persistent dysfunction of HBV-specific T cells (Chen et al., 2007; Kassel et al., 2009). PD-1 is predominantly present on activated T and, B cells, and macrophages, whereas PD-L1 is expressed on tumor cells and activated APCs (e.g., B and T cells, and macrophages, etc.) (Raziorrouh et al., 2014; Hollander et al., 2018; Toor et al., 2019). In chronic viral infections, CD4^+^ T cells undergo dysfunction, and the inactivation of these cells proves inadequate in sustaining CTL function. An effective antiviral response by CD8^+^ T cells necessitates the assistance of CD4^+^ T cells (Brooks et al., 2005; Trautmann et al., 2014).

Recently, immune checkpoint inhibitors (ICIs) have become widely utilized for mitigating T-cell exhaustion in patients with both tumors and chronic infections. ICIs reactivate T-cell-mediated immune responses or release immunosuppression by blocking the IRs pathway, reversing immune escape, and regaining anti-tumor effects and even antiviral effects. Several studies have confirmed the safety of using ICIs to treat patients with HCC and viral hepatitis (Hagiwara et al., 2022; Zhao et al., 2022; Dong et al., 2023; Hagiwara et al., 2023). The combined blockade of multiple IR pathways exhibits better reversal of T cell exhaustion and restoration of effective immune effects, such as PD-1+CTLA-4 (Duraiswamy et al., 2013), PD-1+LAG-3 (Woo et al., 2012), PD-1+TIM-3 (Fourcade et al., 2010; Sakuishi et al., 2010), and PD-1+LAG-3+CTLA-4 (Berrien-Elliott et al., 2013). Blocking the PD-1/PD-L1 pathway helps to restore the capacity of depleted CD8^+^ T cells to proliferate and secrete the pro-inflammatory cytokines IFN-γ, IL-2, and IL-12 (Muthumani et al., 2011; Wherry and Kurachi, 2015; Tang et al., 2016). Additionally, as novel targeted therapies, T cell receptor-engineered T cell (TCR-T) and chimeric antigen receptor T cell (CAR-T) therapies have shown promising potential in HBV immunotherapy, offering effective strategies to address immune deficiencies and dysfunctions in CHB.

#### 2.5.2 MAIT cell

In addition to dysfunctional effector T cells, various other T cell types also undergo immune failure. Mucosal-associated invariant T (MAIT) cells, identified as CD161TCR iVα7.2 T cells, constitute one of the most prevalent populations of innate-like T cells in humans. Exhibiting enrichment in the liver, skin, and mucosal barriers, MAIT cells are restricted by the nonclassical MHC-1b molecule, MR1, executing a vital function in innate defense and countering bacterial infection (Le Bourhis et al., 2013; Yong et al., 2018). In a recent study involving patients with chronic HBV infection, it was observed that the MAIT cells were diminished in number, and there was a remarkable increase in the expression of CD57, PD-1, TIM-3, and CTLA-4 on these cells. Additionally, MAIT cells in patients with chronic HBV infection exhibit lower levels of granzyme (Gr)B and IFN-γ production compared to healthy subjects. Moreover, the peripheral blood MAIT cells are both reduced in number and functionally impaired in patients with chronic HBV infection. The findings indirectly imply that MAIT cells can potentially contribute to the regulation of HBV replication, implying that immune exhaustion of MAIT cells might hinder HBV clearance (Yong et al., 2018). However, considering that TCR Vα7.2 MAIT cells are prominently concentrated in the liver and mucosal tissues of healthy patients (Dusseaux et al., 2011), as an innate T cell, MAIT plays a pivotal role, and future studies are expected to aid in virus clearance by improving MAIT cell functionality. This enhancement is expected to contribute to a more effective clearance of the virus. SsRNA40, a Toll-like receptor 8 agonist, has been employed to trigger the expression of IL-12 and IL-18 in hepatic monocytes to indirectly activate hepatic MAIT cells. In both healthy livers and livers infected with chronic virus (HBV or HCV), it can be detected that ssRNA 40-induced MAIT cells selectively produce heightened levels of IFN-γ, showing positive therapeutic significance for treating chronic liver infection (Jo et al., 2014). In addition, resting human MAIT cells lack GrB and have a low perforin expression of perforin, a key granule protein required for effective cytotoxic activity. MAIT cells maintain high expression of GrB and perforin when stimulated by antigens and cytokines, indicating that these cells can rapidly generate cytotoxic responses (Kurioka et al., 2015) and secrete antiviral cytokines like IFN-γ and TNF-α (Le Bourhis et al., 2013; Godfrey et al., 2015). The human liver has a network of immune cells that regulate immune responses to various pathogen-associated molecules. Consequently, future studies are encouraged to reevaluate the immunotherapeutic potential of activating innate immune cells within the liver.

#### 2.5.3 The role of NK cells

Patients with CHB are enriched in NK cells expressing TNF-related apoptosis-inducing ligand (TRAIL). Heightened levels of TRAIL’s death receptor, TRAIL-R2, are a feature of intrahepatic CD8^+^ T cells in HBV infection. Activated NK cells that possess an elevated level of TRAIL serve a dual function during the infection. They induce apoptosis in HBV-specific CD8^+^ T-cells, thereby dampening the immune response against the virus. Additionally, they promote the death of hepatocytes, which could potentially contribute to liver damage (Dunn et al., 2007; Peppa et al., 2013). Previous research found that the up-regulation of TRAIL-R2 elevates the sensitivity of T cells to apoptosis mediated by caspase-8. TRAIL-R2 sends apoptosis signals to most liver CD8^+^T cells. Additionally, it was observed that blocking TRAIL overnight can partially save the apoptosis of HBV-specific T cells in the liver (Peppa et al., 2013). A recent review revealed that B- and NK-cells have an immunosuppressive effect on CD8^+^ T cells in patients with CHB, inducing apoptosis of CD8^+^ T cells through TRAIL/TRAIL-R2 interactions and inhibiting CD8^+^ T cell function via Gal-9/TIM-3 interactions. Immunomodulatory NK cells (NK-reg) are capable of suppressing the growth of CD8^+^ T cells and secretion of IFN-γ in an IL-10-dependent process, while immature B cells inhibit CD8^+^ T cell responses by producing IL-10 (Allahmoradi et al., 2023). Furthermore, Bregs cells producing IL-10 can enhance the function of Tregs and inhibit effector T cell function. This suggests a correlation between Bregs activity and effector T cell exhaustion (Liu et al., 2016).

#### 2.5.4 The role of pro-inflammatory cytokines

Certain cytokines contribute to the process of T-cell exhaustion. In addition to IL-10, other cytokines contributing to T-cell exhaustion include IL-6, TGF-β, and TNF-α. Notably, IL-10 and TGF-β are implicated in promoting PD-1 expression on the surface of T-cells and contribute to the induction of T-cell exhaustion (Tauriello et al., 2018; Jimbu et al., 2021; Kuo et al., 2021). Cytokines that antagonize T-cell exhaustion include IL-2 and IL-12 (Halwani et al., 2008; Abbas et al., 2018; Kalia and Sarkar, 2018). Depleted T cells are unable to be activated by proinflammatory cytokines but could be co-stimulated with IL-12 and cognate peptides. Although blocking co-inhibitory signals such as PD-1 and CTLA-4 can lead to partial restoration of the functional effects of specific CD8^+^ T cells (Barber et al., 2006; Kaufmann et al., 2007; Wang et al., 2007), the addition of IL-12 further improves the depleted T cell function. A previous study demonstrated that the addition of IL-12 to the SIV DNA vaccine increases the functional effects of effector memory CD8^+^ T cells and promotes IFN-γ/TNF-α production (Halwani et al., 2008). Due to their ability to stimulate proliferation and effector differentiation, IL-2 and IL-12 have therapeutic potential to restore depleted T cell function over the duration of chronic HBV infection. Therefore strong and sustained IL-2 and IL-12 signaling may be considered for immune activation interventions targeting chronic HBV infection.

## 3 Differential expression of major transcription factors such as T-bet and Eomes

T-bet is an identified transcription factor essential for the development or differentiation of Th1, CD8^+^ T, and B cells, and certain innate lymphocyte populations in response to antigens. T-bet can regulate a network of genetic programs, thereby broadly regulating the transcriptional program. This includes coordinating the differentiation, function, migration, and survival of effector and memory lymphocyte subpopulations within the immune system (Lazarevic et al., 2013; Kallies and Good-Jacobson, 2017). T-bet and eomesodermin (Eomes) are central transcription factors that modulate CD8^+^ T-cell exhaustion and memory fates (Doering et al., 2012). Specifically, T-bet and Eomes regulate dysfunctional CD8^+^ T cell responses upon viral infection (Intlekofer et al., 2007; Joshi et al., 2007; Intlekofer et al., 2008; Banerjee et al., 2010; Hersperger et al., 2011; Kao et al., 2011) and appear to be closely associated with the differentiation and self-renewal of memory CD8^+^ T cells (Intlekofer et al., 2007; Joshi et al., 2007; Banerjee et al., 2010). Upon chronic viral infection, virus-specific CD8^+^ T cells express a heightened level of Eomes and a reduced level of T-bet. This inverse expression pattern also represents a balance between naïve memory and transitional frequencies of memory CD8^+^ T cells upon chronic viral infection, which contributes to the hypofunction of human virus-specific CD8^+^ T cells. This pattern also maintains persistent and partially effective CD8^+^ T cell responses (Paley et al., 2012; Buggert et al., 2014). Additionally, several studies have confirmed the link between T-bet deficiency and T-cell exhaustion upon chronic viral infections. Human PD-1 is encoded by the PDCD1 gene and T-bet can inhibit Pdcd1 transcription by direct binding to its upstream regulatory elements, thereby making T-bet a direct transcriptional repressor of PD-1. In a study on lymphocytic choroid plexus meningitis virus (LCMV) infection in mice, sustained antigenic stimulation and high viral loads downregulated the T-bet expression in CD8^+^ T cells. This study not only confirms direct inhibition of PD-1 expression by T-bet, but also observes that upon chronic infection, T-bet-deficient antigen-specific CD8^+^ T cells are defined by expression changes of other IRs (e.g., Tim-3, Lag-3, KLRG-1, and CD160) (Kao et al., 2011). Patients with chronic viral infections experience a progressive loss of systemic CD8^+^ T cell immune responses, with a subsequent accumulation of highly depleted T cells in the liver, which is closely related to the imbalance between the self-renewal and terminal differentiation of virus-specific CD8^+^ T-cells. Nevertheless, the effector function of these cells can be partially restored by blocking negatively transduced signals such as PD-1 and Tim-3 (Nakamoto et al., 2008; McMahan et al., 2010; Paley et al., 2012). In summary, T-bet regulates the expression of multiple IRs in depleted CD8^+^ T cells under sustained antigenic stimulation. T-bet and Eomes are intrinsically involved in mediating exhaustion, memory, and differentiation of viral-specific CD8^+^ T cells, especially upon chronic infection, while the absence of major transcription factors may cause greater exhaustion of viral-specific CD8^+^ T cells.

An upregulated expression of T-bet in immune cells also modulates the production of IFN-γ and cytotoxic molecules in effector CD8^+^ T cells. T-bet deficiency is more commonly seen in patients with chronic infections (Kurktschiev et al., 2014). In a previous study on chronic HBV infection (Schurich et al., 2013), the third signaling factor IL-12 induces T-bet to downregulate PD-1 expression and restores IFN-γ production and cytotoxicity in HBV-specific CD8^+^ T cells. Another study found that inducible stimulation with antigen and IL-2 partially restores the function of dysfunctional T-bet-deficient CD8^+^ T cells. To restore effective IFN-γ responses, it is necessary to provide supplementary stimulation with IL-12. This additional stimulation selectively induces the phosphorylation of transcription activator 4 (Stat4) in CD8^+^ T cells expressing T-bet (Kurktschiev et al., 2014). Notably, the presence of T-bet appears to sensitize these T cells to IL-12, possibly by inducing the IL-12Rβ2 expression (Liao et al., 2011). A related study showed that the presence of Stat4 is essential for T-bet in order to execute the complete IL-12-dependent development of Th1 cells, throughout which T-bet functions as a “master regulator” of the Th1 phenotype (Thieu et al., 2008). Med1(Mediator complex subunit 1) plays a critical role in regulating the differentiation of effector CD8^+^ T cells. In Med1-deficient CD8^+^ T cells, transcriptional programs mediated by T-bet and Zeb2 are impaired; however, overexpression of T-bet can rescue the differentiation and survival of these effector cells (Jiao et al., 2022). The resulting data demonstrated the vital function of restoring the immune function of T-bet-deficient CD8^+^ T cells for viral clearance.

The above-mentioned studies focus on the exhaustion and memory fate of CD8^+^ T cells throughout the viral infection, providing new insights and therapeutic potential for optimizing depleted T-cell function to control persistent viral infections. Our review supported the critical roles of transcription factors such as T-bet for viral clearance and argued that their deficiency may be an important mechanism underlying chronic HBV infection. Numerous research studies have pointed out that T-bet deficiency is more chronic and evolutionary, therefore restoring the robust immune function of CD8^+^ T cells by regulating the expression of key transcription factors such as T-bet may be a promising therapeutic target in treating chronic viral infections and immune-related diseases such as cancers.

## 4 Current HBV-related immunotherapy strategies

### 4.1 T-cell engineering

TCR-T cell therapy is a form of adoptive immunotherapy that uses genetic engineering to modify patient T cells with tumor antigen-specific TCRs, enabling precise recognition and targeting of tumor cells. This approach is particularly suited for patients lacking tumor-specific T cells. In recent years, TCR-T therapy has become a research focus and is regarded as one of the most promising immunotherapeutic strategies. The application of HBV-specific TCR-redirected T (HBV-TCR-T) cellular immunotherapy has been seen in clinical practice, specifically in preventing the reemergence of HCC following liver transplantation (Qasim et al., 2015; Hafezi et al., 2021). Furthermore, over-transfer of mRNA HBV-specific TCR-electroporated T cells to HBV-infected human hepatic chimeric mice prompts a gradual decrease in viraemia. This process, however, simultaneously induces limited, transient hepatic inflammation and cellular damage (Kah et al., 2017). Similarly, adoptive transfer of engineered T cells expressing HBsAg or HBcAg-specific TCR into humanized mice infected with HBV, in combination with the viral entry inhibitor myrcludex B, ensures long-term control of HBV infection (Wisskirchen et al., 2019). In a phase I pilot study involving eight patients with HBV-associated advanced HCC, the safety and tolerability of HBV-TCR-T cellular immunotherapy have been primarily observed. This was particularly evident in patients with advanced HBV-HCC but without prior liver transplantation. Of the eight patients, seven demonstrated decreased or stable serum HBsAg levels, and three showed tumor shrinkage. This antitumor activity and antiviral efficacy may be attributed to HBV-TCR-T cells specifically targeting HBV-expressing metastatic tumor cells (Meng et al., 2021). However, this immunotherapy has significant limitations: in chronic HBV-infected patients with HCC, both normal HBV-infected hepatocytes and HBV-DNA-integrated HCC cells express HBV antigens. Targeting these normal infected hepatocytes may lead to severe liver injury, thereby increasing the risk of extrahepatic inflammatory events (Bertoletti et al., 2015; Tan and Schreiber, 2020). Moreover, longer follow-ups are needed in a larger sample size to observe the potential risk of HBV-TCR-T cell immunotherapy on tumor metastasis and/or bleeding as well as its safety and efficacy. In parallel, CAR-T therapy represents another innovative targeted cancer treatment that utilizes genetic engineering to equip T cells with chimeric antigen receptors (CARs), allowing precise recognition and attack of tumor cells. T cells expressing HBV envelope protein-specific CARs localize and act within the liver, effectively and rapidly inhibiting HBV replication. Animal studies indicate that this process induces only transient liver injury (Krebs et al., 2013); however, these results may not fully translate to humans. In clinical applications, the primary adverse effects of CAR-T cell therapy are cytokine release syndrome (CRS) and neurotoxicity. CRS is triggered by extensive CAR-T cell activation of the immune system and subsequent release of inflammatory cytokines, posing life-threatening risks if not promptly managed. Therefore, close monitoring is essential following CAR-T cell infusion. Therefore, in clinical practice, it is essential to account for the potential risk of acute or chronic hepatitis induced by adoptive T-cell therapy.

### 4.2 PD-1 inhibitors

Numerous studies have demonstrated the efficacy of blocking PD-1 (Fisicaro et al., 2010), CD244 (Raziorrouh et al., 2010), CTLA-4 (Schurich et al., 2011), and Tim (Sakuishi et al., 2010) in restoring virus-specific CD8^+^ T-cell immune responses. However, PD-1 pathway blockade therapy dominated in terms of the expression level of IRs and the effect of blockade response (Bengsch et al., 2014). A phase Ib study utilizing PD-1 inhibitors has confirmed the safety and efficacy of nivolumab monotherapy. Among the twelve patients under monotherapy with nivolumab, 11 CHB patients presented a reduction in HBsAg levels, with one even displaying sustained loss of HBsAg (Gane et al., 2019). Blockage of the PD-1 and PD-L pathway could potentially revive the exhausted subpopulation of CD8^+^ T cells selectively. The majority of CD8^+^ T cell subsets that respond well to PD-1 blocking therapy are those at a lesser degree of differentiation, indicating a proliferation explosion. However, the response from more differentiated and exhausted subsets of CD8^+^ T cells remains unsatisfactory (Blackburn et al., 2008; Im et al., 2016). In conclusion, the combined blockade therapy of multiple IR pathways may be more beneficial for rescuing the function of exhausted T cells. Designing strategies to rescue the subgroups of exhausted CD8^+^ T cells that are intolerant to PD-1 blockade is an important target for the future.

### 4.3 TLR agonist

Agonists that target innate immune receptors like TLR7, TLR8, and TLR9 can potentiate type I interferon responses, thereby improving the compromised virus-specific immune functions associated with chronic HBV infection. Stimulation *in vitro* using the TLR8 agonist GS-9688 (Selgantolimod) prompted the expression of IFN-γ and TNF-α by NK cells, and elevated the proportion of IFN-γ-expressing HBV-specific CD8^+^ T cells in approximately half the CHB patients (Amin et al., 2021). The TLR7 agonist (GS-9620) was used to stimulate human PBMCs. GS-9620 achieved a long-term inhibition of HBV in human hepatocytes through a Type I interferon-dependent mechanism, and it also enhanced the antigen presentation of HBV (Niu et al., 2018). Another clinical trial corroborated the influence of GS-9620 on the reactivity of HBV-specific T cells and NK cells in patients with chronic HBV infection (Boni et al., 2018). Agonists of TLR7/8/9, novel immunomodulators in the oral delivery form, are under preclinical or clinical development. However, limited studies have yet to observe these drugs’ efficacy in reducing serum HBsAg levels.

### 4.4 Therapeutic strategies for B cell dysregulation in CHB

Dysfunction of B cells can be addressed through targeted therapies to restore their virus-specific responses. Salimzadeh et al. showed that in CD40L-expressing feeder layer cells, B-cell maturation cytokines, like IL-2 and IL-21, can partially restore HBsAg-specific B-cell maturation. Furthermore, they observed that the addition of a PD-1 blocker further promotes the functional recovery of dysfunctional HBsAg-specific B cells and their ability to secrete anti-HBs (Salimzadeh et al., 2018). Although the PD-1/PD-Ls complex is an inhibitor of the B-cell activation cascade, deregulation of immunosuppression by Toll-like receptor 9 (TLR9) agonists blocking the PD-1/PD-Ls pathway enhances B-cell activation, proliferation, and inflammatory cytokine production (Thibult et al., 2013). The resulting data on virus-associated B-cell exhaustion provide potential targets for ameliorating impaired B-cell responses during persistent viral infection.

## 5 A potential antiviral therapy: HBV entry inhibitor

NTCP has been identified as the key receptor facilitating the entry of HBV and its satellite virus, HDV, into hepatocytes (Goutam et al., 2022). Inhibiting NTCP expression can prevent HBV invasion and block the initiation of the viral life cycle. The pre-S1 domain of the HBV large envelope protein binds to NTCP on the hepatocyte membrane, mediating viral attachment and entry, underscoring NTCP’s critical role in HBV infection (Li et al., 2024). Given that NTCP function is regulated by complex mechanisms, exploring combination therapies involving immunotherapies and NTCP inhibitors is essential. Some drugs can directly inhibit the invasion and infection of liver cells by HBV through antiviral mechanisms that target NTCP and reticulin. To date, numerous anti-HBV entry inhibitors have been identified, including Myrcludex-B, bile acids, cyclosporine, ezetimibe, ritonavir, irbesartan, and vanillin A (Iwamoto et al., 2014; König et al., 2014; Ni et al., 2014; Nkongolo et al., 2014; Watashi et al., 2014; Yan et al., 2014; Ko et al., 2015; Veloso Alves Pereira et al., 2015). All of these inhibitors target NTCP, with Myrcludex B approved for HDV treatment in Europe since 2020 (Kang and Syed, 2020). *In vitro* studies demonstrate that cyclosporine A effectively blocks HBV and HDV entry by inhibiting the NTCP (Nkongolo et al., 2014). Initial data indicate that a dosage of 2 mg/day of Myrcludex B (Bulevirtide) in combination with TDF demonstrates good safety, tolerability, and efficacy in chronic hepatitis D patients undergoing treatment for 24 weeks or longer (Herta et al., 2022). The results of a Phase Ib/IIa study indicate that Myrcludex B, a novel entry inhibitor, combined with pegylated interferon α-2a, reduces HDV-RNA levels in chronic hepatitis D patients after 24 weeks of treatment (Bogomolov et al., 2016). This drug combination demonstrates a strong synergistic antiviral effect on both HDV-RNA and HBV-DNA. Natural products represent a significant source for new drug discovery. Proanthocyanidin (PAC) is a polymer flavanol extracted from grape seeds, identified as a potential inhibitor of HBV entry. PAC targets amino acids 2-48 in the preS1 region of the large HBV surface protein, thereby preventing HBV from entering host hepatocytes by obstructing the interaction between HBsAg and NTCP (Tsukuda et al., 2017). *In vitro* experiments conducted by Tsukuda et al. demonstrated that PAC reduced HBV infection in a dose-dependent manner (Tsukuda et al., 2017). Primary human hepatocytes were pretreated with various concentrations of PAC (2.5, 5, 10, 20, 40, and 60 μM) for 24 hours, followed by HBV inoculation for 16 hours. After washing away free HBV and compounds, the cells were further cultured for 12 days. Negative controls consisted of untreated cells, while positive controls were treated with PreS1 peptide, a known HBV entry inhibitor. Results indicated that the minimum effective concentration of PAC was 5 μM, with half-maximal inhibitory concentration (IC50) and cytotoxic concentration (CC50) of approximately 7.8 ± 0.75 μM and <80 μM, respectively. Additionally, long-term treatment (39 days) with 3 μM PAC exhibited clear anti-HBV activity in primary human hepatocytes without cytotoxicity. In conclusion, the entry of HBV into hepatocytes is a critical factor in initiating and proliferating infection, rendering viral entry a promising target for antiviral treatment. The clinical value of HBV entry inhibitors has been well established.

## 6 Summary and outlook

Persistent HBV infection disrupts the homeostasis of both humoral and cellular immunity, resulting in immune dysfunction in both B cells and T cells. On one hand, patients with CHB exhibit diminished HBsAg-specific B cells and compromised antibody secretion. The downregulation of various co-stimulatory molecules on B cells affects the coordinated activation of both B and T cells, resulting in an attenuated cytotoxic response of CTLs and indicating B cell dysfunction. On the other hand, the release of IL-10 by Bregs contributes to a negatively regulated immune response. Co-expression of multiple IRs, persistent viral antigenic stimulation, and key transcription factors such as T-bet and Eomes can influence the exhaustion, memory, and differentiation fates of virus-specific CD8^+^ T cells. The differential expression patterns of these factors may contribute to the dysfunction observed in exhausted CD8^+^ T cells. Virus-specific CD8^+^ T cells also exhibit an imbalance between self-renewal and terminal differentiation. These multiple factors functioning together could cause T cell exhaustion and affect the mutual synergy between B cells and T cells. Both B cell subsets and T cell subsets fail to exert their respective HBV-specific immune effects, ultimately manifesting as T-B immune tolerance. However, the exact transcriptional mechanism underlying the regulation of CD8^+^ T-cell differentiation and exhaustion in HBV infection remains unclear. Further elucidating how crucial sets of transcription factors, such as T-bet and Eomes, collaboratively impact the development of virus-specific CD8^+^ T cells, will be a crucial avenue for future research.

This review examines the relationship between T and B cells during chronic HBV infection and surveys related research on immunomodulatory strategies, including the use of immunomodulators and therapeutic vaccines. It is exciting that a variety of new immunotherapies are being developed, offering novel approaches to restore or enhance adaptive immunity. Based on recent advancements in immunotherapy strategies for CHB, future research should focus on overcoming T and B cell immune tolerance, restoring effective immune interactions and activation between B cells and T cells, and identifying new therapeutic targets to enhance the immune functions of both cell types. NUCs and interferons have significant limitations, including inadequate viral suppression, drug resistance, and adverse drug reactions, which hinder the achievement of a functional cure. However, certain HBV entry inhibitors demonstrate promising clinical effects, suggesting that a combined strategy of immunotherapy and new antiviral drugs may offer a more effective solution. We aim to reactivate the host’s adaptive immune response, stimulate specific B cell and T cell responses, inhibit HBV entry through a novel antiviral pathway, and ultimately achieve continuous HBV control via immune activation to promote a functional cure.
